# ﻿Two new *Ptychoptera* Meigen, 1803 (Diptera, Ptychopteridae) from the Western Palaearctic

**DOI:** 10.3897/zookeys.1166.96193

**Published:** 2023-06-06

**Authors:** Libor Dvořák, Katarína Fogašová, Jozef Oboňa, Edina Török, Peter Manko

**Affiliations:** 1 Tři Sekery 21, CZ-35301 Mariánské Lázně, Czech Republic Unaffiliated Mariánské Lázně Czech Republic; 2 Department of Ecology, Faculty of Humanities and Natural Sciences, University of Prešov, 17. novembra 1, SK-08116 Prešov, Slovakia University of Prešov Prešov Slovakia; 3 “Lendület” Landscape and Conservation Ecology Research Group, Institute of Ecology and Botany, Centre for Ecological Research, Alkotmány str. 2-4, Vácrátót, H-2163, Hungary Institute of Ecology and Botany, Centre for Ecological Research Vácrátót Hungary

**Keywords:** Balkan Peninsula, Caucasus, distributional data, new species, phantom crane flies, Ptychopteridae

## Abstract

*Ptychopteraxanthopleura* Dvořák, Oboňa & Manko, **sp. nov.** from Azerbaijan and Georgia, and *Ptychopterastaryi* Dvořák, Oboňa & Manko, **sp. nov.** from Bulgaria are described. *P.xanthopleura***sp. nov.** differs from the other member of the *lacustris* group mainly by having almost completely yellow pleurae, and by the shape of the epandrium and gonocoxites. The diagnostics of *P.staryi***sp. nov.** and *P.incognita* Török, Kolcsár & Keresztes, 2015 based on male genitalia are provided.

## ﻿Introduction

Ptychopteridae comprises two extant subfamilies: Bittacomorphinae Shiner and Ptychopterinae Alexander. Bittacomorphinae is found in the Nearctic, East Palearctic, and Oriental regions (e.g., Nakamura and Saigusa 2009). Ptychopterinae is widely distributed in the world except for the Australasian Region (Hancock et al. 2006; Fasbender 2017).

The genus *Ptychoptera* Meigen, 1803 comprises more than 90 recent species worldwide (e.g., Kang et al. 2013, 2019, 2022; Fasbender 2014; Zhang and Kang 2021), in Europe one genus occurs, namely *Ptychoptera*, with 18 species (Oosterbroek 2006; Török et al. 2015; Keresztes et al. 2021).

During the last ca. 40 years, eight new *Ptychoptera* were described from the Western Palaearctic region; one from Italy (*P.delmastroi* Zwick & Starý, 2003), four from the Balkans and neighbouring areas (*P.agnes* Krzemiński & Zwick, 1993 from Hungary, *P.incognita* Török, Kolcsár & Keresztes, 2015 from Romania and Bulgaria, and *P.castor* Keresztes & Kappert, 2021 and *P.pollux* Keresztes & Török, 2021 both from the south Balkan area), and three from the Caucasus (*P.peusi* Joost, 1974 from Russian Caucasus, *P.ressli* Theischinger, 1978 from Iran, and *P.alina* Krzemiński & Zwick, 1993 from Armenia).

The adults live in marshy and moist habitats, near suitable substrates for the larvae as well as quite some distance from the larval habitat (Oosterbroek 2006). The larvae are aquatic to semi-aquatic, living in muddy water, shallow pools in marshes, *Sphagnum* pools, or the margins of streams (e.g., Andersson 1997; Wiberg-Larsen et al. 2021).

Our present study reports the description of two new *Ptychoptera* species, one from the Balkans and one from the Caucasus.

## ﻿Materials and methods

### ﻿Taxonomic material

The terminology of male genitalia follows Fasbender (2014) and Kang et al. (2022); the other morphological terminology adopted here follows essentially that of Zitek-Zwyrtek (1971), McAlpine (1981), and Oosterbroek (2006).

All voucher specimens are deposited and accessible in the private collection of the 1^st^ author. Genitalia of *Ptychopterastaryi* sp. nov. and *P.xanthopleura* sp. nov. were macerated in 10% KOH and dehydrated using a series of dehydrating alcoholic solutions (70%, 80%, 96%). After that, parts of genitalia were mounted on permanent slides using Canada balsam as mounting medium.

Photographs of specimens were taken using a Pentax K-50 camera and a reverse-mounted Vivitar 28 mm 1: 2.0 MC lens, Motic SMZ-1 68 stereomicroscope equipped with Canon EOS 1200D camera and EOS utility software, and with Leica M205C stereomicroscope equipped with Leica DFC295 digital camera. Focus stacking was performed using Adobe Photoshop.

### ﻿Phylogenetic analysis in the case of *P.xanthopleura* sp. nov.

We undertook phylogenetic analyses to understand the relationships between the species in the subgenus Paraptychoptera. Cladistic analyses of 53 morphological characters on antennae, wing, and male terminalia (Table 1) were selected based on Keresztes et al. (2021) and Fasbender (2014). The list of morphological characters is described in detail by Keresztes et al. (2021).

**Table 1.** Matrix of the 53 morphological data (based on the Keresztes et al. 2021) used in the phylogenetic analyses. For description of characters and character states, see Keresztes et al. (2021). ’?’ – missing data.

	**1**	**2**	**3**	**4**	**5**	**6**	**7**	**8**	**9**	**10**	**11**	**12**	**13**	**14**	**15**	**16**	**17**	**18**	**19**	**20**	**21**	**22**	**23**	**24**	**25**	**26**	
***P.contaminata***	0	0	0	0	0	0	0	0	0	0	0	0	0	0	0	0	0	0	0	0	0	0	0	0	0	0	
*P.agnes*	?	1	1	1	?	?	?	1	1	0	0	1	1	1	0	0	0	0	0	0	1	0	1	1	0	0	
*P.castor*	1	1	1	1	0	1	0	1	0	0	0	1	0	0	0	0	0	0	0	1	1	0	1	0	0	1	
*P.delmastroi*	?	?	?	?1	1	0	1	1	0	1	0	1	0	0	0	0	0	1	0	0	1	1	0	0	0	0	
*P.handlirschi*	1	1	1	1	1	0	1	1	0	1	0	1	1	0	1	0	0	0	0	0	1	1	0	0	0	0	
*P.helena*	1	1	1	1	0	1	0	1	0	0	0	1	0	0	0	0	0	0	1	0	1	0	1	0	1	0	
*P.lacustris*	1	1	1	1	0	1	0	1	0	0	0	0	0	0	0	0	0	0	0	0	1	1	0	0	0	0	
*P.longicauda*	1	1	1	1	1	0	1	1	0	1	0	1	0	0	0	0	0	1	0	0	1	1	0	0	0	0	
*P.paludosa*	1	1	1	1	1	0	1	1	0	1	0	1	1	0	0	1	0	0	0	0	1	1	0	0	0	0	
*P.pollux*	1	1	1	1	0	1	0	1	0	0	0	1	0	0	0	0	0	0	1	0	1	0	1	0	0	0	
*P.resseli*	?	?	?	?	0	?	?	1	0	0	1	1	0	0	0	0	0	1	0	0	1	0	1	1	0	0	
*P.silvicola*	1	1	1	1	1	0	1	1	0	1	0	1	1	0	0	0	1	0	0	0	1	1	0	0	0	0	
*P.xanthopleura* sp. nov.	1	1	1	1	1	1	0	1	0	1	0	0	0	0	0	0	0	0	0	0	1	0	1	1	0	0	
	**27**	**28**	**29**	**30**	**31**	**32**	**33**	**34**	**35**	**36**	**37**	**38**	**39**	**40**	**41**	**42**	**43**	**44**	**45**	**46**	**47**	**48**	**49**	**50**	**51**	**52**	**53**
***P.contaminata***	0	0	0	0	0	0	0	0	0	0	0	0	0	0	0	0	0	0	0	0	0	0	0	0	0	0	0
*P.agnes*	0	1	1	0	1	0	1	1	0	0	1	1	0	0	0	1	1	1	1	0	1	1	0	0	1	0	1
*P.castor*	0	1	1	1	0	0	1	0	1	0	1	0	0	0	1	1	1	1	0	1	1	0	1	0	1	0	1
*P.delmastroi*	0	1	1	0	0	0	1	1	0	0	0	0	1	0	0	1	1	1	1	0	1	1	0	0	1	1	0
*P.handlirschi*	0	1	1	0	0	0	1	1	0	0	0	0	1	0	0	1	1	1	1	0	1	1	0	0	1	1	0
*P.helena*	0	1	1	1	0	0	1	0	1	0	1	0	0	0	1	1	1	1	0	1	1	0	1	0	1	0	1
*P.lacustris*	0	1	1	0	0	0	1	0	1	0	1	0	0	1	0	1	1	1	1	0	1	0	1	0	1	0	1
*P.longicauda*	0	1	1	0	1	0	1	0	0	1	1	1	0	0	0	1	1	1	1	0	1	0	1	0	1	1	0
*P.paludosa*	0	1	1	0	0	1	1	1	0	0	0	0	1	0	0	1	1	1	1	0	1	1	0	0	1	1	0
*P.pollux*	1	1	1	1	0	0	1	0	1	0	1	0	0	0	1	1	1	1	0	1	1	0	1	0	1	0	1
*P.resseli*	0	1	1	0	0	0	1	1	0	0	0	0	0	1	0	1	1	1	1	0	1	0	0	1	1	1	0
*P.silvicola*	0	1	1	0	0	0	1	1	0	0	0	0	1	0	0	1	1	1	1	0	1	1	0	0	1	1	0
*P.xanthopleura* sp. nov	0	1	1	1	0	0	1	0	1	0	1	1	0	0	1	0	1	1	0	1	1	1	1	0	1	0	1

The morphological data matrix was created and managed with Mesquite 3.5 (Maddison and Maddison 2019) and analysed using Maximum parsimony and Bayesian analysis. For maximum parsimony analysis, we used TNT “Tree Analysis using New Technologies” v. 1.5 (Goloboff and Catalano 2016). A “traditional” search based on 1000 replicates of Wagner trees, through ‘tree bisection reconnection’ (TBR) branch swapping holding 100 trees by the collapsing rule: ‘min. length=0’. Subsequently, we selected the best tree, in terms of species topology and population phylogeographical clades, and resampled with 100 000 replicates using a standard bootstrap procedure. Values at nodes represented absolute frequencies and frequency differences (GC, Group present/Contradicted).

For visualisation of the phylogenetic tree, we used FigTree v. 1.4.4 (http://tree.bio.ed.ac.uk/software/figtree/), and the character state and statistical values visualisation were plotted onto the trees using Adobe Photoshop.

## ﻿Taxonomic account

### ﻿Genus Ptychoptera

Taxon classificationAnimaliaDipteraPtychopteridae

Meigen, 1803

25F17EBD-5D5F-5900-9DEC-424D79C48F18

#### Diagnosis.

Medium sized to large (7–15 mm), slender, black lustrous Nematocera, often with lighter markings on thorax and/or abdomen. Antennae, wings, abdomen, and legs long and slender. Ocelli absent; antenna with 15 to 16 segments. Thorax with deep, posteriorly directed transverse suture. Wing with markings, in particular along the crossveins and where veins bifurcate; spurious vein present on either side of crossvein R-M and wing membrane with a distinct fold between veins A_1_ and C_u_A_2_ (Oosterbroek 2006; Fasbender 2014).

### ﻿ Ptychoptera xanthopleura

Taxon classificationAnimaliaDipteraPtychopteridae

Dvořák, Oboňa & Manko sp. nov.

4ABF56F3-B29C-529A-85B5-F704180E8F15

https://zoobank.org/CD413B9C-177D-4A4C-ACB9-3565C502C27B

Figs 1 , 2

#### Type material.

***Holotype***: 1 ♂: Azerbaijan, Qum, sidebrook/small tributary of the Ardavacaj (Ardavachay) River + wetland, 845 m a. s. l., 41°28'10.3"N, 46°55'57.2"E, 8.V.2019, leg. J. Oboňa & P. Manko. ***Paratypes***: 2 ♂♂: Georgia, border of Imereti and Samtskhe–Javakheti regions, brook and spring, south slope of Zekari pass, 2 050 m a. s. l., 41°49'23"N, 42°51'09"E, 17.VII.2019, leg. G. Vinçon.

#### Description.

**Male. *Head***: Frons, vertex, and occiput black with metallic blue shine, mouthparts including palpi pale yellow, scape and pedicel yellowish orange, antennal flagellomeres a somewhat darker, tending to pale brown.

***Thorax***: Scutum, paratergite, and mediotergite blackish with metallic blue shine; scutellum, pleurotergite, katepisternum, and katepimeron brownish black with lighter metallic blue shine; other parts yellow. Halteres yellow with light brown knob. Legs yellow except brown extreme apex of femora and tibiae, tarsi somewhat darkened.

**Figure 1.**
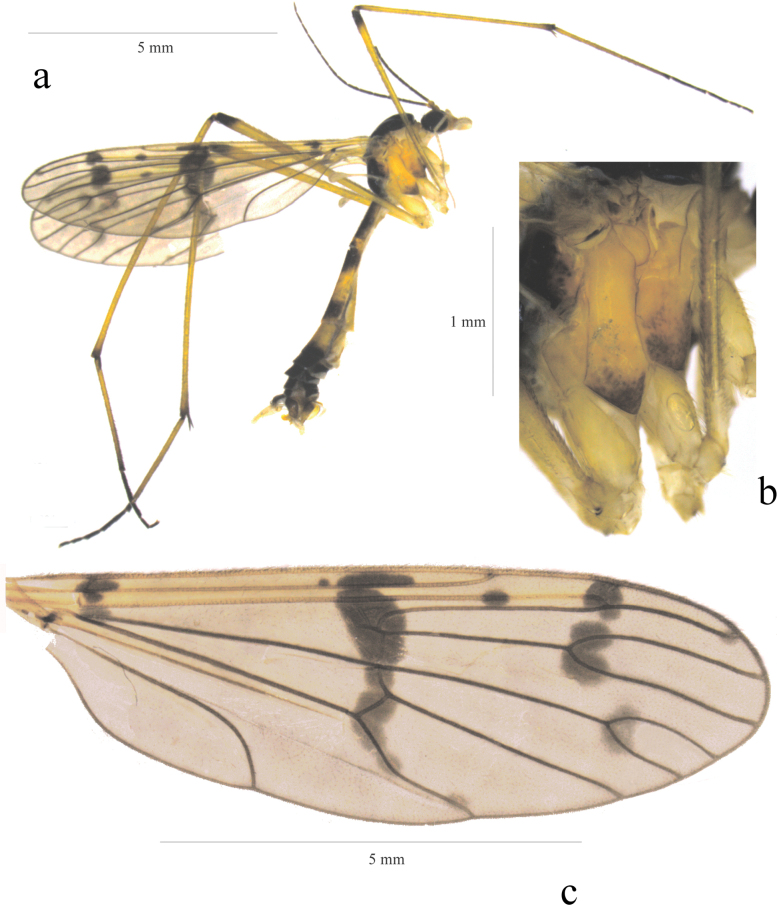
*Ptychopteraxanthopleura* sp. nov. **a** habitus of male, lateral view **b** pleurae, lateral view **c** wing.

Wing length 10 mm (holotype, Fig. 1c). Wing almost hyaline, veins yellowish brown, distinct spots brownish black, forming more or less three stripes, at base of wing at the level of crossvein h, from C to Cu. Middle stripe touching vein C, running through cross-veins up to middle part of vein Cu, isolated spot before end of vein Cu. Isolated spot on around middle of R_1_. Third stripe consist of three large, almost touching spots: at the tip of R_1_ and fork of R_2_+_3_, one on fork vein R_4_+_5_ and one on fork vein M_1_+_2_. Small spot at the end of vein R_3_.

***Abdomen***: Tergum 1 dark shiny brown with yellow apex, sternum 1 yellow. Tergum 2 brown basally and apically, yellow in middle, sternum 2 yellow. Tergum 3 yellow basally, brown apically, sternum 3 yellow. Remaining terga and sterna brown, sternum 4 yellow basally. Auxiliar copulatory organ yellow.

**Figure 2.**
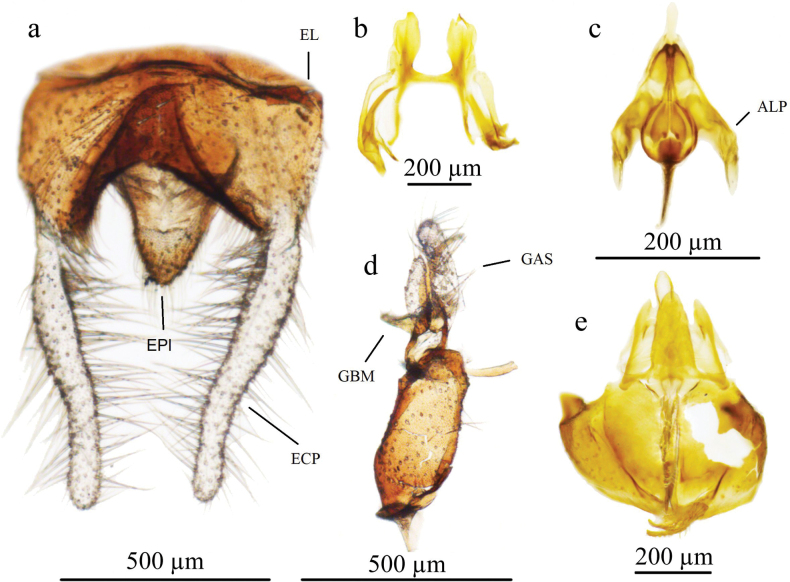
*Ptychopteraxanthopleura* sp. nov. terminalia **a** epandrium **b** paramere **c** aedeagal complex **d** gonostylus **e** hypandrium. Abbreviations: ECP = epandrial clasper, EL = epandrial lobe, EPI = epiproct, GBM = medial lobe of basal lobe of gonostylus, GAS = apical stylus of gonostylus, ALP = lateral ejaculatory process.

***Male genitalia*** (Fig. 2): Hypopygium almost 2× as wide as long, widest in basal quarter, medially with very deep emargination. Epandrial claspers simple and long (length/width ratio ca. 5.8) and covered by long pale hairs, the longest hair up to 1.75 longer than width of epandrium. Apical stylus of gonostylus robust and rounded apically, secondary lobe long, reaching almost 0.75 of apical stylus length. Media lobe of basal lobe of gonostylus long and sharply pointed (saber-like); anterior lobe of basal lobe of gonostylus bulbous apically with several setae at extreme apex.

**Female.** The authors have an immature female which was sampled in Lesser Caucasus (Georgia, Kakheti region, Ilto river, above (N of) the Chart’ala village, 790 m a. s. l., 42°8'18"N, 45°7'32"E, 8.VII.2019, leg. P. Manko & G. Vinçon). The characters correspond to the above-described new species. However, its identity cannot be confirmed in this stage of ontogenesis/development and could be solved after collecting more specimens of the genus *Ptychoptera* from the Transcaucasia.

#### Etymology.

The name reflects predominantly yellow pleurae (Fig. 1b), which are unique for the Western Palaearctic species.

#### Differential diagnosis.

**Figure 3.**
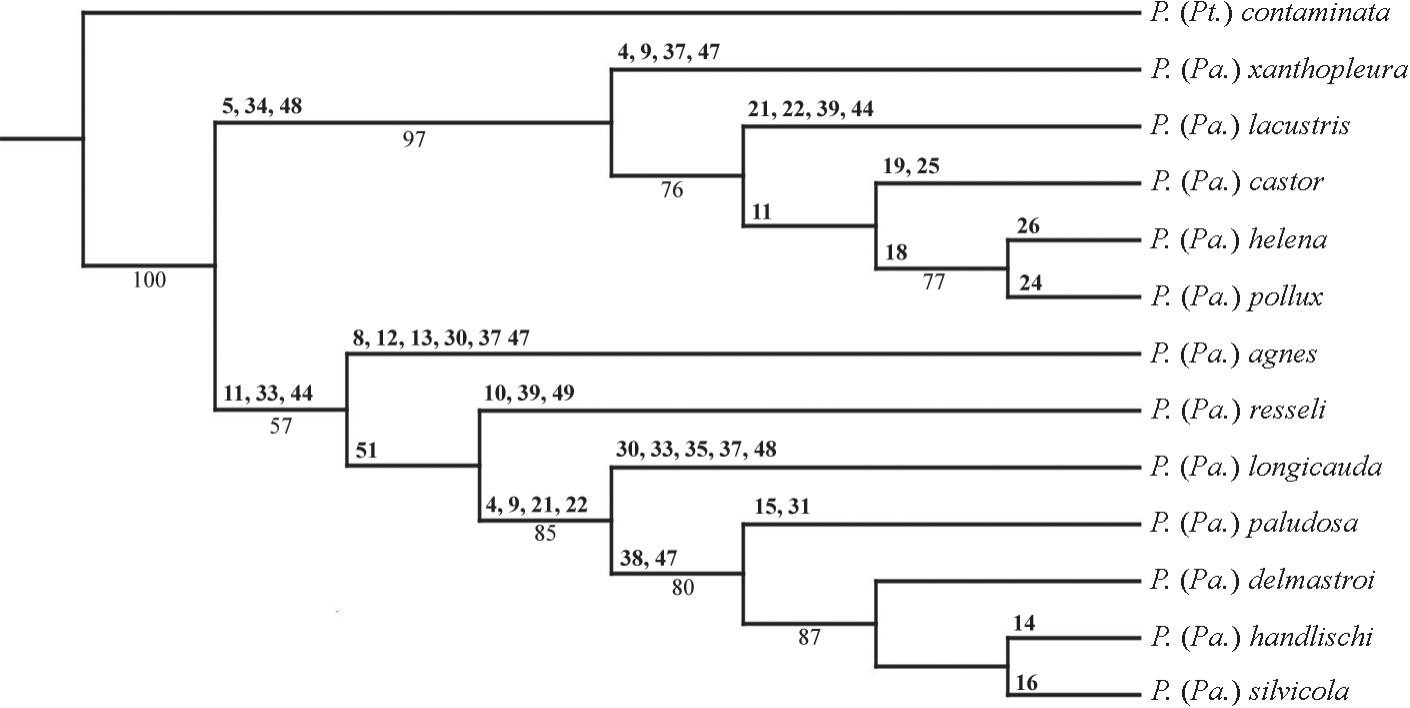
The maximum parsimonious tree based on 53 morphological characters. The characteristics are shown above branches. The parentheses show the maximum parsimony phylogeny bootstrap support value expressed as percentages. Support values under 40 are not shown.

According to the presence of auxiliary sexual organ and shining pleurae, *P.xanthopleura* sp. nov. belongs to the subgenus Paraptychoptera and according to male genitalia and the maximum parsimonious tree based on 53 morphological characters (see Fig. 3 and Table 1), the nearest species is *P.lacustris* and belongs to the highly divergent monophyletic unit, the *lacustris* group, including five species, *P.xanthopleura* sp. nov., *P.lacustris*, *P.castor*, *P.helena*, and *P.pollux* (Fig. 3). *Ptychopteraxanthopleura* sp. nov. is close to but differs from the most similar species *P.lacustris* mainly by having an almost completely yellow pleurae, the shape of the hypopygium (epandrial claspers, secondary lobe of gonostylus, and medial lobe of basal lobe of gonostylus).

### ﻿ Ptychoptera staryi

Taxon classificationAnimaliaDipteraPtychopteridae

Dvořák, Oboňa & Manko sp. nov.

BFF28901-CF56-5393-9BCF-41D24D443143

https://zoobank.org/21E4C563-97EA-497A-92C6-02A6A8621746

Figs 4 , 5

#### Type material.

***Holotype***: 1 ♂: Bulgaria, Rhodopes, Yundola, 1 300 m a. s. l., 42°3'47"N, 23°51'17"E, 30.VI.2016, leg. M. Barták et Š. Kubík.

#### Description.

**Male. *Head***: Frons, vertex, and occiput black, mouthparts including palpi pale yellow, scape and pedicel yellowish orange, antennal flagellomeres greyish.

***Thorax***: Predominantly black with silvery pubescent pleurae. Pronotum, epimeron 3 and metanotum 3 yellow. Fore and mid coxae and trochanters yellow, hind coxae brownish black basally, yellow apically, coxae also yellow. Almost all legs are missing. Halteres whitish yellow with a darker knob.

**Figure 4.**
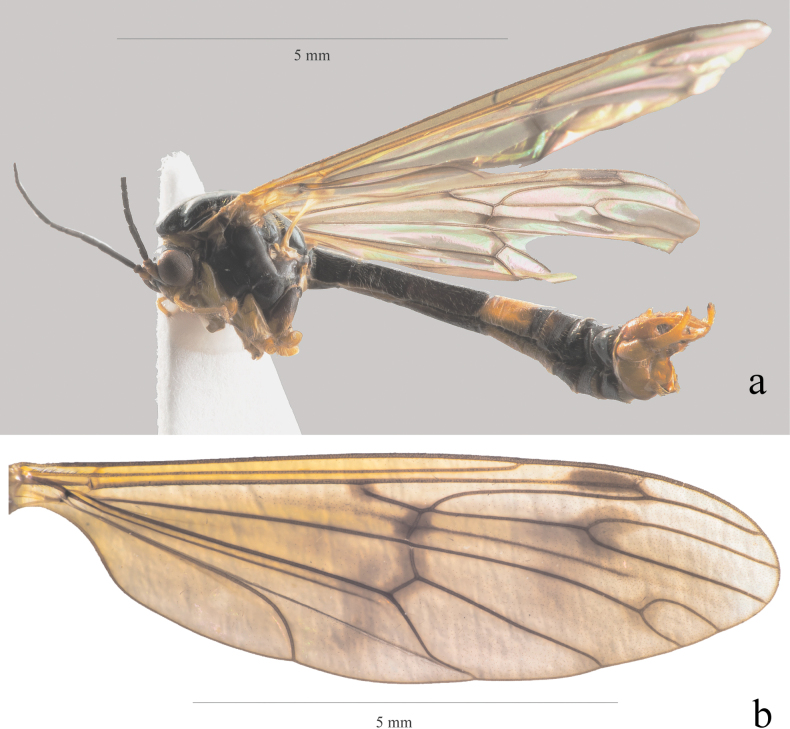
*Ptychopterastaryi* sp. nov. **a** habitus of male, lateral view **b** wing.

Wing length 12 mm (Fig. 4b). Wing with yellowish veins and infuscated spots on fork vein Rs+R, all cross-veins, end of R_1_ vein up to its fork with R_2_ vein, on fork vein R_4_+R_5_ and on fork vein M_1_+M_2_. Legs: femur pale, darker in apical ¹⁄5, almost black on extreme apex; tibia pale brown in basal ½, darker apically and almost black on extreme apex; tarsomere 1 almost black, tarsomeres 2 and 3 dark brown, tarsomeres 4 and 5 pale brown.

***Abdomen***: Tergum 1, tergum 2, apical ¹⁄5 of tergum 3, almost whole tergum 4 except base, and whole terga 5–7 black; sternum 1 black, sternum 2 brown, sterna 5–7 black; the remainder orange yellow.

***Male genitalia***: similar to *P.incognita*. Epandrial clasper slightly curved outwards with simple obtuse apex; anterior projection of ventromesal lobe sharp, posterior projection bow-shaped backwards; space between both projections is rounded, almost semi-circular. Gonocoxite and gonostylus: apical process of paramere with a U-shaped dark structure with thick edges; paramere base rounded, convex; width to height ratio of dorsal gonocoxal lobe 0.5; dorsal gonocoxal lobe with dense tiny dark hairs. Aedeagus: sides of lateral ejaculatory process distinctly convex, basal projections markedly convergent; transition to lateral ejaculatory process smooth, undulate. Hypandrium: width to length ratio 1.2; apex of hypogynial valves start under basal division of hypandrium. See also differential diagnosis.

**Female.** Unknown.

#### Etymology.

The name is dedicated to our colleague Jaroslav Starý and his life jubilee. (Jaroslav discovered the holotype of new species in his own material and provided it for the description).

#### Differential diagnosis.

**Figure 5.**
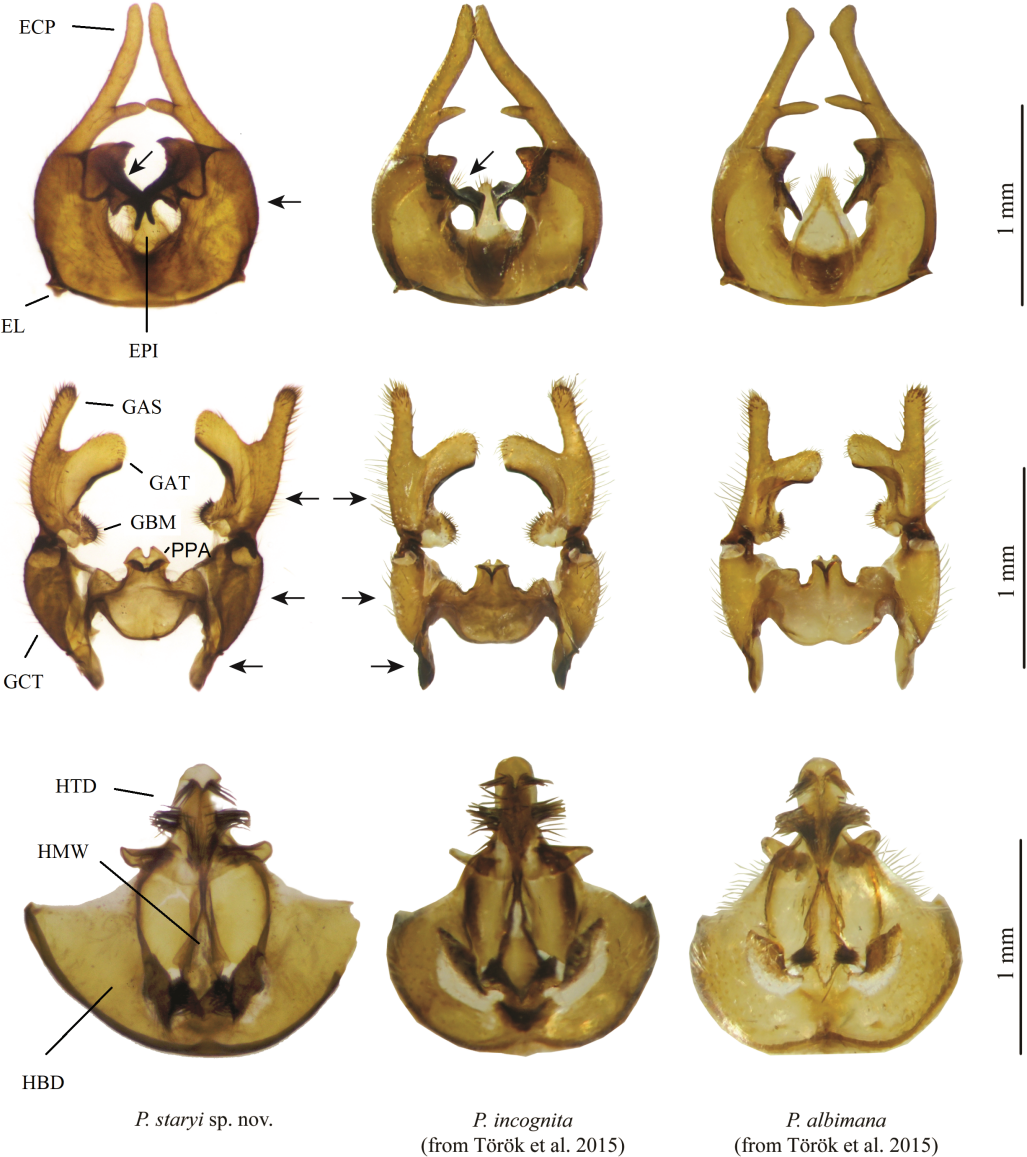
Epandrium, gonocoxite and gonostylus, and hypandrium of *P.staryi* sp. nov., *P.incognita*, and *P.albimana.* Abbreviations: ECP = epandrial clasper, EL = epandrial lobe, EPI = epiproct, GBM = medial lobe of basal lobe of gonostylus, GCT = gonocoxite, GAS = apical stylus of gonostylus, GAT = tertiary lobe of apical stylus of gonostylus, PPA = apical process of paramere, HBD = basal division of hypandrium, HMW = membranous window of terminal division of hypandrium, HTD = terminal division of hypandrium.

The new species is very similar to *P.incognita* Török, Kolcsár & Keresztes, 2015 (see also Table 2). After comparing the holotype of *P.staryi* sp. nov. with individuals of *P.incognita* (material used in Török et al. 2015: 2 individuals from Bulgaria, 17 individuals from Romania), we found that a diagnosis was possible on the basis of differences in male genitalia (Fig. 5, marked with arrows), namely: (i) shape of the plate on ventral parts of epandrium is of a different shape and orientated at a different angle in *P.staryi*; (ii) chitinisation (sclerotisation) of the proximo-lateral processes of the gonocoxite is not developed and these processes are light coloured in *P.staryi* in contrast to *P.incognita* with strong chitinisation and dark colouration; (iii) the hairs of margins of gonocoxite, gonostylus, and epandrium are much less dense and finer in contrast to *P.incognita* (not visible in Fig. 5).

**Table 2.** Diagnostics of *P.staryi* sp. nov., *P.incognita*, and *P.albimana* based on male genitalia (see also Fig. 5).

	*P.staryi* sp. nov.	*P.incognita*	*P.albimana*
Epandrium	Shape of the plate on ventral site and the space between projections rounded, almost semi-circular.	Projections of the plate on ventral site in about right angle, space between projections quadrangular.	Projections of the plate on ventral site in obtuse angle, space between projections rounded, more than semicircle.
Gonocoxite and gonostylus	Width to height ratio of dorsal gonocoxal lobe 0.5; dorsal gonocoxal lobe with dense tiny dark hairs. The hairs of margins of gonocoxite and gonostylus much less dense and finer. Chitinisation of the proximo-lateral processes of the gonocoxite not developed, processes light coloured.	Width to height ratio of dorsal gonocoxal lobe 0.6; dorsal gonocoxal lobe with sparse small hairs. The hairs of margins of gonocoxite and gonostylus dense and stronger. Chitinisation of the proximo-lateral processes of the gonocoxite strongly chitinised, dark.	Width to height ratio of dorsal gonocoxal lobe 0.4; dorsal gonocoxal lobe with tiny dark hairs at the top. The hairs of margins of gonocoxite and gonostylus dense and stronger. Chitinisation of the proximo-lateral processes of the gonocoxite not developed, processes pale coloured.
Aedeagus	Sides of lateral ejaculatory process distinctly convex, basal projections markedly convergent; transition to lateral ejaculatory process smooth, undulate.	Sides of lateral ejaculatory process slightly convex, basal projections divergent; transition to lateral ejaculatory process steep, in a right or acute angle to the axis of aedeagus.	Sides of lateral ejaculatory process slightly convex, basal projections almost parallel; transition to lateral ejaculatory process steep, in an obtuse angle to the axis of aedeagus.
Hypandrium	Width and length ratio 1.2; apex of hypogynial valves start under basal division of hypandrium.	Width and length ratio 0.9; apex of hypogynial valves start on bases of basal division of hypandrium.	Width and length ratio 0.9; apex of hypogynial valves start over basal division of hypandrium.

## Supplementary Material

XML Treatment for Ptychoptera

XML Treatment for Ptychoptera xanthopleura

XML Treatment for Ptychoptera staryi

## ﻿Additional information

### Author ORCIDs

Libor Dvořák https://orcid.org/0000-0002-4712-3679

Katarína Fogašová https://orcid.org/0009-0003-9604-1885

Jozef Oboňa https://orcid.org/0000-0002-1185-658X

Edina Török https://orcid.org/0000-0001-5982-7078

Peter Manko https://orcid.org/0000-0003-1862-9117

### Data availability

All of the data that support the findings of this study are available in the main text.
